# A siRNA-Based Screen for Genes Involved in Chromosome End Protection

**DOI:** 10.1371/journal.pone.0021407

**Published:** 2011-06-23

**Authors:** Daniel H. Lackner, Daniel Durocher, Jan Karlseder

**Affiliations:** 1 Molecular and Cellular Biology Department, The Salk Institute for Biological Studies, La Jolla, California, United States of America; 2 Samuel Lunenfeld Research Institute, Mount Sinai Hospital, Toronto, Ontario, Canada; Tulane University Health Sciences Center, United States of America

## Abstract

Telomeres are nucleoprotein complexes which protect the ends of linear chromosomes from detection as DNA damage and provide a sequence buffer against replication-associated shortening. In mammals, telomeres consist of repetitive DNA sequence (TTAGGG) and associated proteins. The telomeric core complex is called shelterin and is comprised of the proteins TRF1, TRF2, POT1, TIN2, TPP1 and RAP1. Excessive telomere shortening or de-protection of telomeres through the loss of shelterin subunits allows the detection of telomeres as DNA damage, which can be visualized as DNA damage protein foci at chromosome ends called TIF (Telomere Dysfunction-Induced Foci). We sought to exploit the TIF phenotype as marker for telomere dysfunction to identify novel genes involved in telomere protection by siRNA-mediated knock-down of a set of 386 candidates. Here we report the establishment, specificity and feasibility of such a screen and the results of the genes tested. Only one of the candidate genes showed a unique TIF phenotype comparable to the suppression of the main shelterin components TRF2 or TRF1 and that gene was identified as a TRF1-like pseudogene. We also identified a weak TIF phenotype for SKIIP (SNW1), a splicing factor and transcriptional co-activator. However, the knock-down of SKIIP also induced a general, not telomere-specific DNA damage response, which complicates conclusions about a telomeric role. In summary, this report is a technical demonstration of the feasibility of a cell-based screen for telomere deprotection with the potential of scaling it to a high-throughput approach.

## Introduction

Telomeres are the physical ends of linear eukaryotic chromosomes. In mammals, telomeres consist of several kilobases of the repetitive DNA sequence TTAGGG that end in a short (50–500 nt) single-stranded overhang, which has the ability to invade the double-stranded telomere sequence to form a lasso-like structure [1]. To ensure proper telomeric function, this DNA structure is additionally bound by telomere-specific proteins. The main complex of these associated proteins is the shelterin complex, which consists of 6 proteins called TRF1, TRF2, RAP1, TPP1, TIN2, and POT1 [2]. TRF1 (Telomeric Repeat Binding Factor 1) and TRF2 (Telomeric Repeat Binding Factor 2) directly bind to the double-stranded repeat region, whereas POT1 (Protection of Telomeres 1) can bind to the single-stranded overhang of the telomeric repeat. TIN2 (TRF1-interacting Nuclear Factor 2) can be seen as a scaffolding unit, as it can bind both TRF1 and TRF2, and also bridges these double-strand binding factors to POT1 via an interaction with TPP1 [2]. Mammalian RAP1 (ortholog of the yeast Repressor/Activator Protein 1) binds to TRF2 [3] and whereas the yeast ortholog is one of the major proteins involved in telomere dynamics, deletion of RAP1 in the mammalian system shows only a weak telomeric phenotype 4,5. However, RAP1 has been reported to have additional, non-telomeric functions in the regulation of transcription and NFkB-signaling [4], [6].

Telomeres serve two main purposes: they act as sequence buffer to counteract replication-associated shortening and they protect the ends of our chromosomes from being recognized as DNA damage. Failure to protect telomeres properly has dramatic consequences for cells and organisms. Critically short or unprotected telomeres are subject to increased levels of recombination, lead to altered gene expression patterns and silencing dysfunctions [1], [7]. In mammals, dysfunctional telomeres are recognized as double strand breaks and processed by the non-homologous end joining (NHEJ) machinery [8]. This leads to fusion of chromosomes, and in the subsequent replication cycles to genome instability, growth arrest or cell death [7]. Unprotected telomeres can arise by defects in the shelterin complex. One of the best examples is disruption of TRF2 through overexpression of a dominant-negative allele or siRNA-mediated knock-down or targeted deletion, which after few generations leads to high amounts of telomeric end-to-end fusions [9], [10], [11], [12], [13].

Another phenotype of telomere dysfunction, and an even earlier sign of telomere de-protection than telomeric fusions, is a localized DNA damage signal at telomeres. This association of DNA damage response factors, such as g*αμμα*-H2AX or 53BP1 with telomeres is termed TIF (Telomere Dysfunction-Induced Focus/Foci) [14]. TIF formation can be due to extensive loss of telomeric sequences or loss of telomere end protection in the presence of double stranded TTAGGG repeats. While TIF indicate the recognition of telomeres as DNA breaks and can lead to a widespread damage response, chromosome fusions or death, the outcome can be much milder and with less consequences, indicating temporary telomere deprotection [15].

These varying potential outcomes suggest that a number of factors play roles in transient or permanent telomere protection and deprotection. In order to identify novel regulators we developed a screen that combines siRNA-mediated knock-down of candidate genes with the use of TIF as read-out of telomere dysfunction. Here, we describe the establishment of such a screen, report on potential pitfalls, and discuss identified candidates and their potential role in protection of telomere integrity.

## Results

Our approach was based on the assumption that factors, whose targeting leads to a TIF phenotype, would be either directly involved in telomere protection or be regulators and modifiers of shelterin components. Furthermore we reasoned that suppression of a gene product that results in TIF formation would also cause a general DNA damage phenotype, when telomeric localization is not considered as defining criteria. We therefore focused on a group of genes that has been selected in a genome-wide approach designed to identify general players in the DNA damage response. When cells are subjected to ionizing radiation they form foci call IRIF (Ionizing Radiation Induced Foci) [16], consisting of proteins involved in damage recognition and repair at sites of lesions. A genome-wide screen designed to identify factors, whose suppression affect IRIF formation after ionizing irradiation [17] allowed us to focus on a list of 520 target factors. These genes haven initially been identified by knock-down using the siGENOMERNAi pools from Dharmacon, but have been further verified using alternative siRNAs by the Durocher laboratory. The shelterin components TRF1 and TRF2 were identified as modifiers of the damage response by the aforementioned screen and therefore contained within this list, suggesting that proteins that play a role in telomere function can be isolated with this approach. We further adapted the initial gene list and excluded 134 genes that would be unlikely directly linked to genome stability and telomere function (e.g. ribosomal proteins), allowing us to focus on 386 potential factors (Table S1).

**Figure 1:**
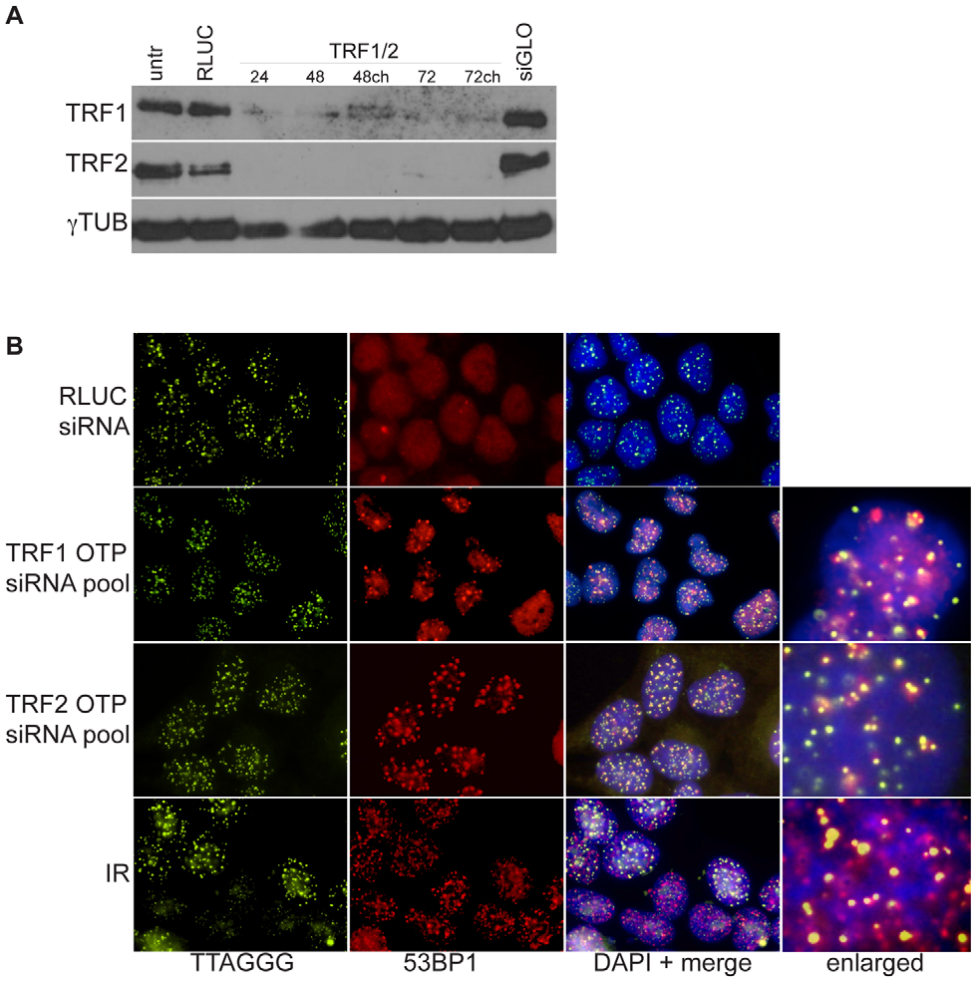
Transfection with siRNA pools reduces TRF1 and TRF2 levels and induces a TIF phenotype. (A) Western analysis of TRF1 and TRF2 expression post transfection with OTP siRNA pools at the indicated time points post transfection. gamma-Tubulin serves as loading control. All knock-downs achieved greater than 80% suppression of the targets. (B) IF-FISH based TIF analysis in cells depleted for TRF1 or TRF2. 53BP1 staining was used as marker for DNA damage and a FITC coupled telomeric FISH probe was used to detect telomeric repeats. Untr: untransfected control, RLUC: control siRNA targeting Renilla luciferase, siGLO: cells transfected with siGLO green transfection indicator, ch: media was changed 24 hours post transfection, IR: cells subjected to 10 Gray of ionizing radiation.

**Figure 2:**
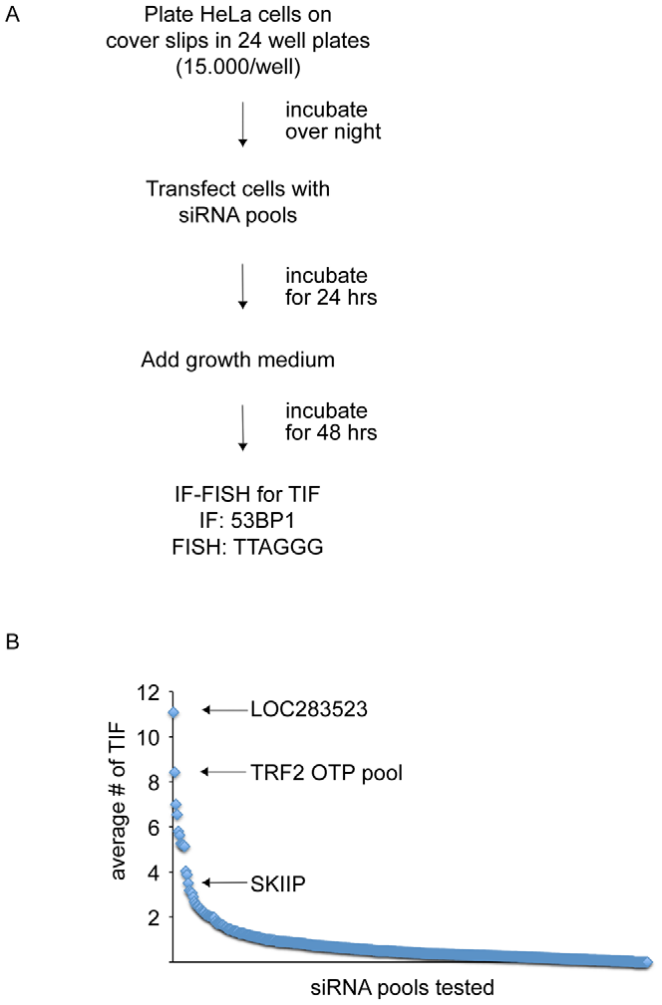
siRNA based TIF screening. (A) Schematic overview of the screen layout. (B) Primary data of all siRNA pools. The positive control (TRF2 OTP siRNA pool), SKIIP and the TRF1 pseudogene LOC283523 are indicated.

We first verified that transfection with according siRNA pools resulted in efficient knock-down of TRF1 and TRF2 and that this suppression was sufficient to induce TIF. For these controls we used the OnTargetPlus (OTP) pools from Dharmacon, the next generation of siRNA pools which differ from the siGENOME pools by a novel target sequence selection algorithm and a unique single-strand RNA modification (http://www.dharmacon.com/product/productlandingtemplate.aspx?id=167&tab=0). Transfection of HeLa 1.2.11 cells with the corresponding siRNA pools substantially reduced TRF1 and TRF2 protein levels already 24 hours post transfection and protein levels remained almost undetectable throughout the whole 72 hour time course (Figure 1A). Knock-down efficiency was unaltered, even if the transfection medium was changed after 24h (Figure 1A). As the siRNA pools for the actual screen were from the siGENOME library from Dharmacon and not the OTP pools, we also verified siRNA-mediated knockdown of TRF1 and TRF2 using siRNA pools from the siGENOME library. For these controls, we used a 24-well setup similar to the one eventually used for the actual screen (outlined in Figure 2A). Transfection with the corresponding siRNA pools reduced TRF1 and TRF2 levels respectively, as evaluated by immunofluorescence (IF) 72h after transfection. Knock-down of TRF2 reduced TRF2 nuclear foci, whereas TRF1 was still clearly recognized in telomeric foci (Figure S1, lower panel). However, knock-down of TRF1 also resulted in a reduced telomeric localization of TRF2 (Figure S1, middle panel). In contrast, total protein levels of TRF2 were not affected by TRF1 knock-down, as has been observed previously (data not shown) [18].

We next tested whether cells transfected with siRNA pools against TRF2 and TRF1 developed TIF as a read out of telomere dysfunction [14]. 72 hours after transfection a clear DNA damage response in the form of 53BP1 foci could be observed in cells transfected with siRNA pools against TRF1 or TRF2, whereas no increase in DNA damage foci was observed in cells transfected with a control siRNA against Renilla luciferase (RLUC) (Figure 1B). Furthermore, the DNA damage foci clearly represented TIF, as they were found almost exclusively at telomeres. The TIF response after TRF2 knock-down was even more pronounced than that after the suppression of TRF1 (Figure 1B). This differs from a general DNA damage response that could be seen when cells are exposed to ionizing radiation (IR), where DNA damage foci were randomly distributed throughout the nucleus and did not co-localize with telomeres (Figure 1B).

Based on these initial experiments we set up our screen as outlined in Figure 2A: HeLa cells were transfected with siRNA pools on coverslips in a 24-well format and fixed in paraformaldehyde 72 hours post transfection. A combination of IF and Fluorescence In Situ Hybridization (IF-FISH) was then used to stain DNA damage foci and telomeres to detect TIF. On each 24-well plate we included a set of 4 controls: untransfected cells and RLUC siRNA as negative controls, TRF2 OTP pool as positive control, and siGLO transfection indicator from Dharmacon to check for successful transfection of the siRNA (see Material and Methods for details).

Here we want to point out that we detected a low number of cells exhibiting clear TIF in both the RLUC siRNA transfected and untransfected controls. Despite the fact that the overall occurrence of TIF was rare in control cells (Table 1), they always appeared in a similar pattern. Within our controls we could detect clusters of cells that exhibited a very low intensity of telomeric FISH signal, which is indicative of short telomeres. Such clusters of cells frequently exhibited a TIF phenotype (Figure S2A). We speculate that these are occurrences of spontaneous TIF that might arise in sub-populations of cells with short telomeres [15]. For our screen we used a clonal HeLa cell line with long telomeres (HeLa1.2.11), however we suggest that some cells in the culture acquire critically short telomeres over time, which attract DNA damage factors.

**Table 1:** List of identified candidate factors.

Candidate genes with an average of more than 3 TIFs				
Screen ID	# TIFs(a)	Stdev(a)	Gene symbol	Remarks
G198	11.1	7.1	LOC283523	TRF1-like pseudogene, 2 of the siRNAs from pool also target TRF1
D83	6.5	11.4	NHP2L1	few cells, many 53BP1 foci
D29	5.6	2.0	RRM1	many 53BP1 foci
G122	5.3	7.7	MGC2494	few cells
G144	5.2	6.6	MGC13125	some cells with many 53BP1 foci
D37	5.1	3.6	PCNA	many 53BP1 foci
D88	4.0	5.0	YAF2	Phenotype not reproduced
D38	3.9	2.6	POLA	many 53BP1 foci
D14	3.2	2.6	SKIIP	many 53BP1 foci
D6	3.1	4.2	SF3A1	many 53BP1 foci
D89	3.1	2.2	DDB1	many 53BP1 foci

Controls (average from 18 individual experiments)				
	# TIF(b)	Stdev(b)		
Untransfected	0.4	0.5		
RLUC siRNA	0.3	0.4		
TRF2 OTP	8.4	1.6		

Shown are candidate gene products from the screen described in this manuscript, whose suppression induced an average of at least 3 TIF per cell (this corresponds to the average number of TIF per cell for all genes tested plus 2 standard deviations).

(a): averages and standard deviations calculated from all cells scored from one knock-down,

(b): averages and standard deviations calculated from the average of 18 individual experiments.

During the actual screen of the 386 candidate factors we determined the number of TIF in up to 196 cells for each individual knock-down. We obtained data for 382 of the 386 genes tested (Table S1), since four candidates failed to deliver interpretable results due to broken cover slips. Stringent criteria were applied to identify genes as potential candidates for TIF formation. We only considered genes that displayed an average of at least 3 TIF per cell, an arbitrary number that corresponds to the average number of TIF per cell for all genes tested plus two standard deviations and led to the identification of 11positive hits only (Figure 2B, Table 1). Furthermore, between 2–3 TIFs/cell the steepness of the curve in Figure 2B increases, which suggested that this is an appropriate point of separation between noise and real hits. As expected from the bias in selection of the initial set of candidate factors, many of the genes tested exhibited a strong DNA damage phenotype after siRNA-mediated knock-down. Also, the suppression of a number of candidates induced cell death, and as such we could not evaluate the phenotype accurately (data not shown). The analysis of the 11 factors with a clear TIF phenotype was independently repeated twice. In the repetitions all of the hits except YAF2 led to reproducible phenotypes (Table S2).

**Figure 3:**
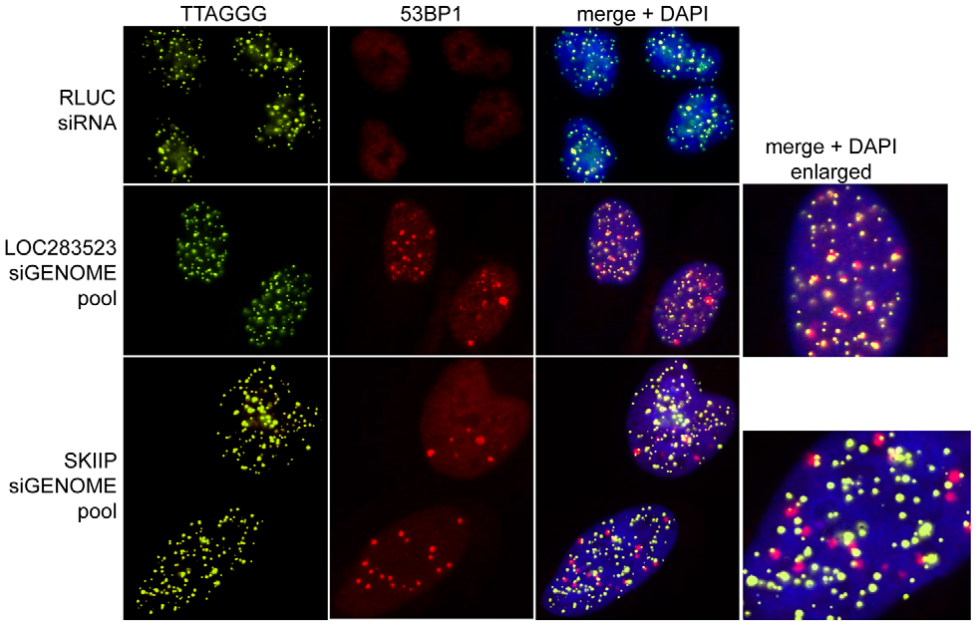
TIF induction by isolated candidate factors. IF-FISH images after the suppression of LOC283523 and SKIIP using the siGENOME pools. RLUC-siRNA transcfected cells serve as control. Telomeres were detected by hybridization to a FITC coupled TTAGGG probe and damage foci were visualized by 53BP1 staining.

Only one target displayed an overwhelmingly TIF-specifc phenotype. Knock-down of LOC283523 induced a strong DNA damage response, and DNA damage focico-localized exclusively with telomeres (Figure 3, middle panels). This gene was also the highest scoring gene in the screen (Figure 2B). However, LOC283523 has been annotated as a TRF1-pseudogene (telomeric repeat binding factor [NIMA-interacting] 1 pseudogene; Gene ID 283523). Due to the high sequence identity of this gene to TRF1, the siRNA pool against LOC283523 also contains 2 siRNAs with a perfect match to the bona fide TRF1 sequence. As a consequence, the observed TIF-phenotype is most likely due to suppression of endogenous TRF1 gene products, supported by the finding that TRF1 expression is found down regulated in cells transfected with LOC283523 siGENOME pool (Figure S2B).

All other candidates listed in Table 1 lead to a weak TIF phenotype, where telomere specific damage foci were observed among a general, non-telomeric DNA damage response. Some of these gene products have well-established roles in DNA metabolism (e.g. RRM1) or replication (POLA, PCNA) and as such it is not surprising that their suppression results in a strong damage response. As a consequence, it is difficult to establish whether the weak TIF phenotype is a direct consequence of the respective knock-down or only occurs as an indirect secondary effect.

**Figure 4:**
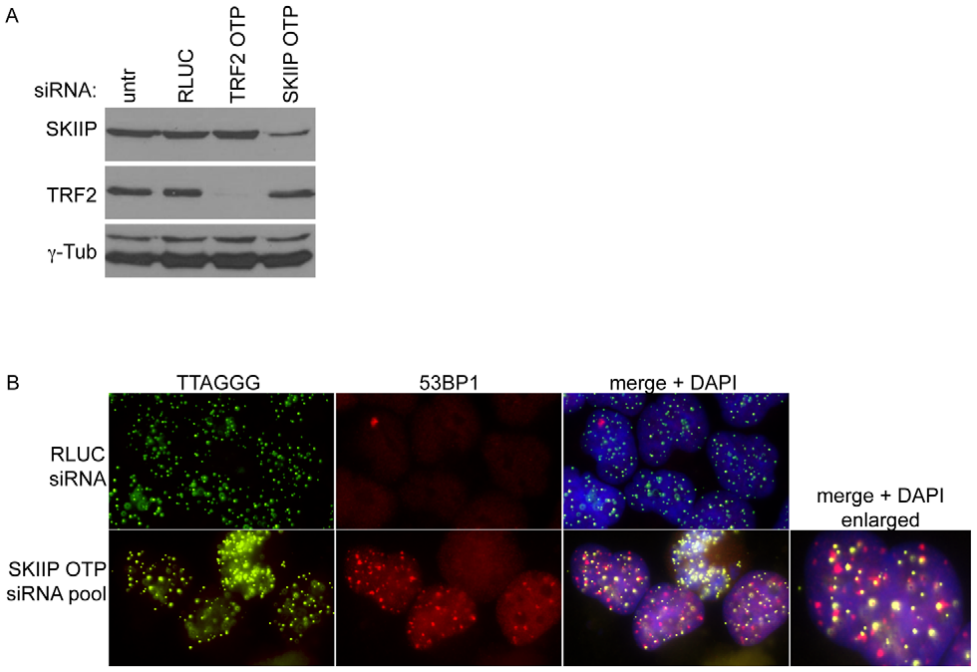
Knock-down of SKIIP using OTP siRNA pools. (A) Western analysis of SKIIP and TRF2 72 hours post transfection with OTP siRNA pools. gamma-Tubulin serves as loading control. All knock-downs achieved greater than 95% suppression of the targets. (B) Visualization of telomeric and non telomeric DNA damage foci in cells depleted for SKIIP using OTP siRNA pools. RLUC-siRNA transcfected cells serve as control. Telomeres were detected by hybridization to a FITC coupled TTAGGG probe and damage foci were visualized by 53BP1 staining.

Within the set of factors leading to a general DNA damage with a weak TIF phenotype we identified several gene products involved in splicing (NHP2L1, MGC13125, SKIIP, SF3A1) (Table 1). Genes associated with splicing and RNA processing have previously been reported to be the most enriched functional group within factors, whose suppression mediates DNA damage [19]. Here we focused on SKIIP (also known as SKIP or SNW1; Gene ID: 22938), since this protein has been shown to associate with telomeres in a large-scale proteomics approach, in which whole telomeres were purified and associated proteins were identified by mass spectrometry [20]. The knock-down of SKIIP by transfection with the corresponding siGENOME siRNA pool induced general DNA damage in the affected nuclei. However, some of the DNA damage foci were localized at telomeres and thus were considered as TIFs (Figure 3, bottom panel). To verify this phenotype, we repeated the suppression using the OTP siRNA pool against SKIIP. SKIIP protein levels were clearly diminished 72 hours after transfection with this siRNA pool (Figure 4A) and we again observed a few, clear TIFs in the background of a general nucleus-wide DNA damage response (Figure 4B).

**Figure 5:**
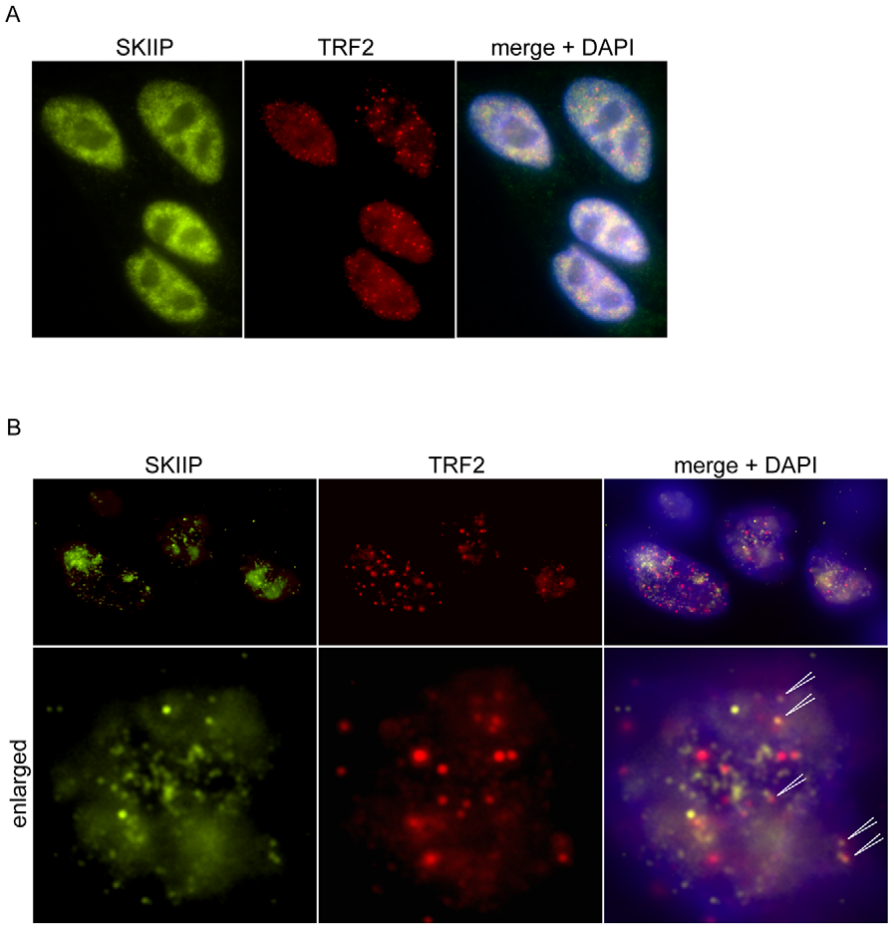
Nuclear localization of SKIIP and TRF2. IF analysis of the nuclear localization of SKIIP and TRF2 in directly fixed (A) or pre-extracted (B) nuclei. Co-localization events in pre-extracted nuclei have been indicated by arrows.

We next tested whether the SKIIP protein can localize to telomeres by immunofluorescence. Cells were stained with an antibody against SKIIP and an antibody against the telomeric marker TRF2. This approach pointed out that SKIIP was present throughout the nucleus, with less intense staining in the nucleolus (Figure 5A) suggesting that the strong expression of SKIIP in the nucleus masked a potential telomere-specific localization. However, when the nuclei were pre-extracted before fixation to remove the soluble protein fraction [21], we could detect distinguished SKIIP foci in the nucleus (Figure 5B) and some of these foci co-localized with telomeric signals (Figure 5B), suggesting that a fraction of SKIIP binds specifically to chromosome ends.

## Discussion

To identify novel players involved in telomere protection we established a siRNA-based screen using TIF as read-out. We demonstrated that the suppression of the shelterin subunit TRF2 using siRNA pools induced a robust TIF response, and we subsequently screened a candidate set of 386 genes, which were known to play roles in DNA damage signaling [17] for TIF induction.

The knock-down of only one gene product (LOC283523) induced a clear and telomere-specific TIF phenotype, however, this factor was identified as a TRF1-pseudogene and the corresponding siRNA pool also targeted TRF1 (Figure S2B). While this factor did not present a novel telomere protection factor, it proved the validity of the screen. Despite this proof of principle we would like to point out some technical limitations of such an approach: First, the occurrences of spontaneous TIF (Figure S2A) [15] create a certain background level of TIF in control cells. These spontaneous TIF occur much more frequently in transformed cell lines than in primary cells. We speculate that these spontaneously de-protected telomeres could result from rare stochastic loss of a protective state at chromosome ends, such as opening of the t-loop. It is also possible that TRF2 is occasionally lost from telomeres to an extent where full protection cannot be achieved anymore, as has been suggested previously [15]. A third possibility would be that these TIF represent a brief cell cycle stage where telomere protection is temporarily lost and chromosome ends are recognized by the DNA damage machinery, despite being fully functional [22]. As primary cell lines are less amenable for large-scale siRNA screens, it might be useful for a screen with higher coverage to identify alternative transformed cell lines with a low background of spontaneous TIF. To avoid this problem during our approach, we applied a stringent cut-off for the identification of positive candidates and only considered siRNAs, which on average induced more than 3 TIF per cell, as compared to the RLUC siRNA control, which only induced an average of 0.3 TIF per cell (averaged from 18 individual knock-downs).

A second problem is the masking of a TIF phenotype by nucleus-wide DNA damage signaling. The suppression of genes involved in DNA metabolism or replication expectedly resulted in a massive DNA damage response, evidenced by numerous nuclear 53BP1 foci. This complicates the identification of TIF, since it increases the likelihood of a coincidental co-localization of DNA damage foci and telomeres.

Third, TIF might also arise as a secondary effect due to general replication defects or widespread genomic instability. While it is possible that some of the players leading to a general damage response have additional and specialized roles at telomeres and that TIF arise independently from the general DNA damage, it is a challenge to discern between these scenarios.

We focused on SKIIP (also known as SNW1 or SKIP), one of our weaker hits, as this protein has been previously reported to bind to telomeres [20]. SKIIP has been shown to act both as transcriptional co-regulator [23], [24], [25], [26], [27], [28] and as component of the splicing apparatus [26], [29], [30], [31]. This dual role made it an interesting candidate for a possible telomeric function, as it might be involved in the regulation of RNA processing of telomeric transcripts (TERRA) and/or might function through its ability to directly bind to chromatin. The suppression of SKIIP with two independent siRNA pools induced a general DNA damage response, but also some clear TIF (Figure 3, Figure 4B). The phenotype was different from spontaneous TIF formed in control cells, as these were mostly seen in cells with low telomeric signal and the DNA damage signal was exclusively telomeric (Figure S2A). Thus, we conclude that the observed phenotype is a specific, albeit weak, telomere damage induction.

SKIIP protein was detected throughout the nucleus, but in pre-extracted nuclei some co-localization events between SKIIP and TRF2 could be observed (Figure 5). However, this result does not allow for a clear conclusion.We cannot exclude a telomere-specific role for SKIIP, but we also cannot rule out telomeric damage signaling simply as a consequence of a secondary indirect effect, as co-localization events with telomeres do not necessarily prove a telomere-specific role for SKIIP. We also found other factors involved in splicing in our set of potential hits (NHP2L1, MGC13125, SF3A1), and splicing factors and RNA processing factors are the most enriched functional group within genes whose knock-down induces DNA damage [19]. It has been speculated that depletion of splicing factors such as SKIIP increases DNA-RNA hybrid formation in the nucleus, which in turn increases recombination events and as a consequence induces DNA damage. In line with this hypothesis, it has been demonstrated that over-expression of RNAse H, which cleaves these DNA-RNA hybrids, alleviates the DNA damage effect induced by depletion of splicing factors such as SKIIP [19].

Since it has recently been reported that telomeres can be transcribed by polymerase II into RNA molecules termed TERRA [32], [33], depletion of splicing factors and resulting problems with co-transcriptional events, such as RNA processing or packaging could lead to detrimental DNA-RNA hybrids and eventually genomic instability at telomeres. Alternatively, depletion of splicing factor might simply interfere with expression levels of a wide variety of genes.

In conclusion, we demonstrated the feasibility, but also the challenges, of using TIF as read-out for a siRNA-based screen to identify novel factors involved in telomere protection. We could successfully validate the approach, and isolated a number of factors with a potential role in telomere function. It is quite likely we missed telomere regulators by limiting our screen to a pre-selected subset of factors. However, performing an unbiased genome wide approach with all 22.000 targets of the currently available genome wide RNAi libraries would require a different experimental setup and a much higher degree of automation, with an emphasis on automatic image recognition of TIF.

## Materials and Methods

### Cell line and culture conditions

All experiments were performed using HeLa 1.2.11 cells, which were grown in DMEM medium (GIBCO, #10569) containing 10% Bovine Growth Serum (Thermo Scientific, #SH30541.03), antibiotics (Cellgro, #30-002-Cl), and non-essential amino acids (Cellgro, #25-025-Cl). Cells were grown at 7.5% CO_2_ and 3.5% oxygen. For transfections with siRNAs, cells were grown in the absence of antibiotics. siRNAs and transfection reagents were diluted in OPTI-MEM (GIBCO, #11058).

### Antibodies

For Western Blotting, in-house made polyclonal antibodies against TRF1 and TRF2 were used; a polyclonal antibody against SKIIP was a gift from the lab of Katherine Jones (Salk Institute). For Immunofluorescence we also used the following primary antibodies: 53BP1 (Santa Cruz, #sc-22760), SNW1 (Sigma Prestige Antibodies, #HPA002457), TRF2 (Millipore, # 05-521). As secondary antibodies, we used anti-mouse or anti-rabbit monoclonal antibodies coupled to Alexa-488 or Alexa-594 (Molecular Probes, Invitrogen). As loading control we used gamma-Tubulin (Sigma, #T6557).

### siRNA screen

Knock-downs were performed using siGENOME smart pools from Dharmacon (Fisher Scientific), OTP plus smart pools (Fisher Scientific) ora siRNA against Renilla luciferase (Fisher Scientific, # P-002070-01-20) as indicated. Transfection efficiency was monitored using siGLO green transfection indicator (Fisher Scientific, # D-001630-01-20).15.000 cells were plated on Alcian blue-treated coverlips in 24-well format the evening before transfection. Transfection with siRNAs was done using Dharmafect 1 according to the manufacturer’s protocol. Per well (500ul total volume), 0.5ul of Dharmafect 1 and a final siRNA concentration of 50nMol was used. 24 hours after transfection an additional 500ul of growth medium was added to dilute the transfection reagent and avoid toxicity. 72 hours after transfection cells were fixed in 2% Paraformaldehyde. To detect TIF, Immunofluorescence and telomere FISH staining (IF-FISH) was performed as previously described [15]. We used 53BP1 as marker for DNA damage and a FITC-coupled telomeric PNA probe (FITC-TelC [FITC-OO-(CCCTAA)3], Applied Biosystems) for telomere FISH. Images were taken on a Zeiss Axio Imager Z1 at 63x magnification. For each condition 1-196 nuclei were scored for the occurence of TIF. For transfections of siRNAs in other than the 24-well format, reagents were scaled up accordingly.

### Immunofluorescence (IF) and Western blotting

IF was performed using the same reagents as for the IF-FISH protocol, but omitting the FISH part. All images were taken at comparable magnifications. Pre-extraction of nuclei was performed as previously described [21]. For whole cell protein extracts, cells were harvested using trypsinization, washed in PBS and then directly suspended in 2xLDS loading buffer according to cell numbers. DNA was sheared using a syringe. Protein extracts were resolved on 10% SDS-PAGE gels, transferred to nitrocellulose, blocked in TBS buffer with 0.5% (w/v) non-fat dry milk and 0.1% (v/v) Tween20 for 1 hour and probed with the according antibodies. As secondary antibodies, HPR-linked anti-rabbit or anti-mouse antibodies were used (GE Healthcare, #NA934V and #NXA931), and the HPR signal was visualized with ECL Western Blotting Substrate (Pierce, #32106) or Supersignal West Pico Sensitivity Substrate (Pierce, #34080).

## Supporting Information

Figure S1 **Knock-down of TRF1 and TRF2 using siGENOME pools.** IF analysis of expression levels of TRF1 and TRF2 72 hours post transfection of corresponding siRNA pools from the siGENOME library. Transfection of RLUC siRNA was used as control.
(TIF)

Figure S2 (A) Spontaneous TIF formation in control cells. IF-FISH analysis of cells in RLUC-transfected control cells. Telomeres were detected by hybridization to a FITC coupled TTAGGG probe and damage foci were visualized by 53BP1 staining. (B) Targeting LOC283523 reduces TRF1 expression. Western analysis of TRF1 expression after transfection with siGENOME pool against LOC283523 72 hrs post transfection.gamma-Tubulin serves as loading control.
(TIF)

Table S1 **List of all candidate genes tested in the screen.** No data were obtained for the genes indicated in red due to technical problems.
(DOCX)

Table S2 **Repetitions of suppression of 11 candidate factors.**
(DOCX)
