# Supplementary figures and images for: A siRNA-Based Screen for Genes Involved in Chromosome End Protection
Source: PLoS One. 2011 Jun 23;6(6):e21407. doi: 10.1371/journal.pone.0021407 (PMC3121770; doi:10.1371/journal.pone.0021407)

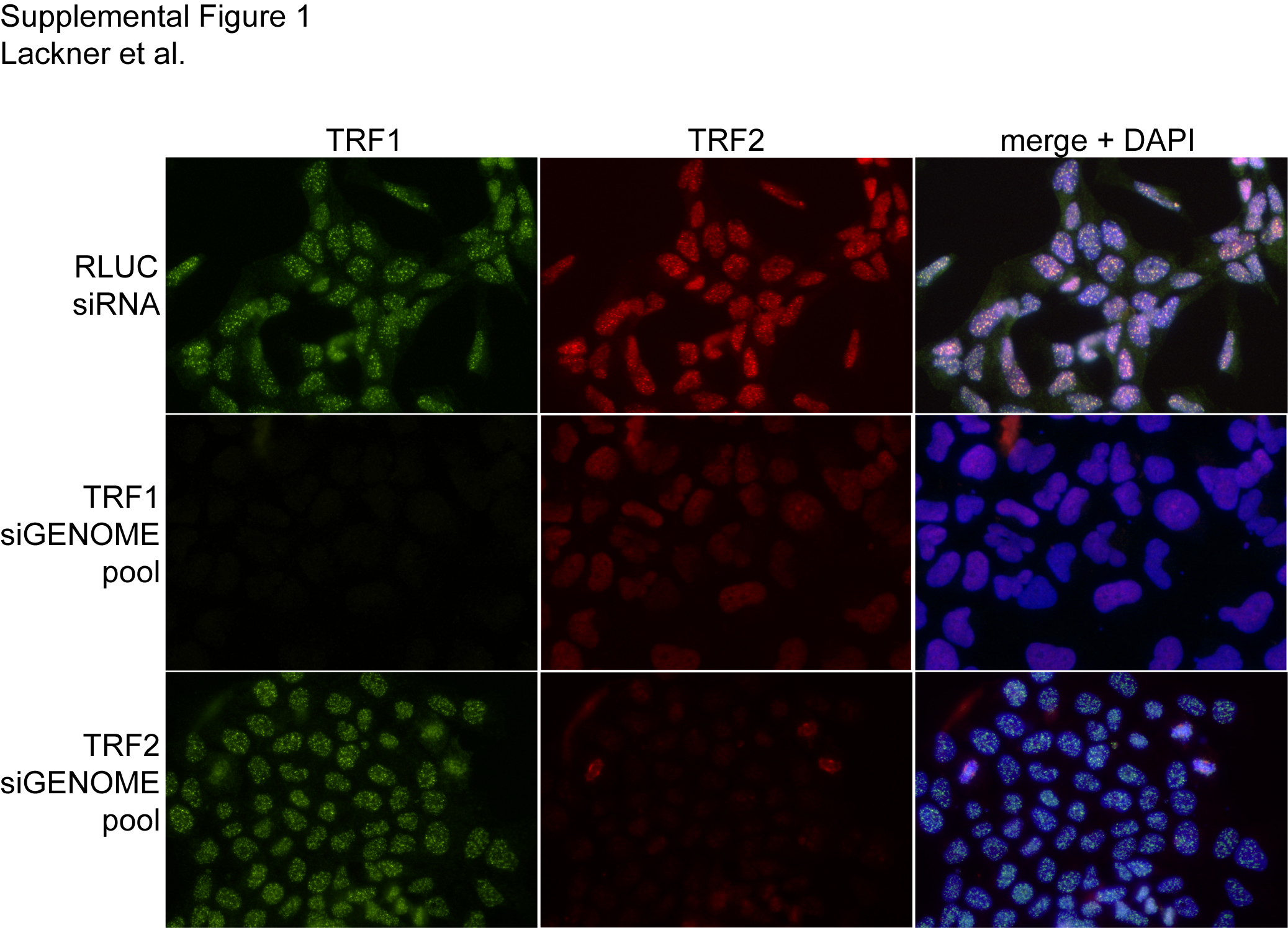

Supplement: Figure S1 — Knock-down of TRF1 and TRF2 using siGENOME pools. IF analysis of expression levels of TRF1 and TRF2 72 hours post transfection of corresponding siRNA pools from the siGENOME library. Transfection of RLUC siRNA was used as control. (TIF) [file pone.0021407.s001.tif]

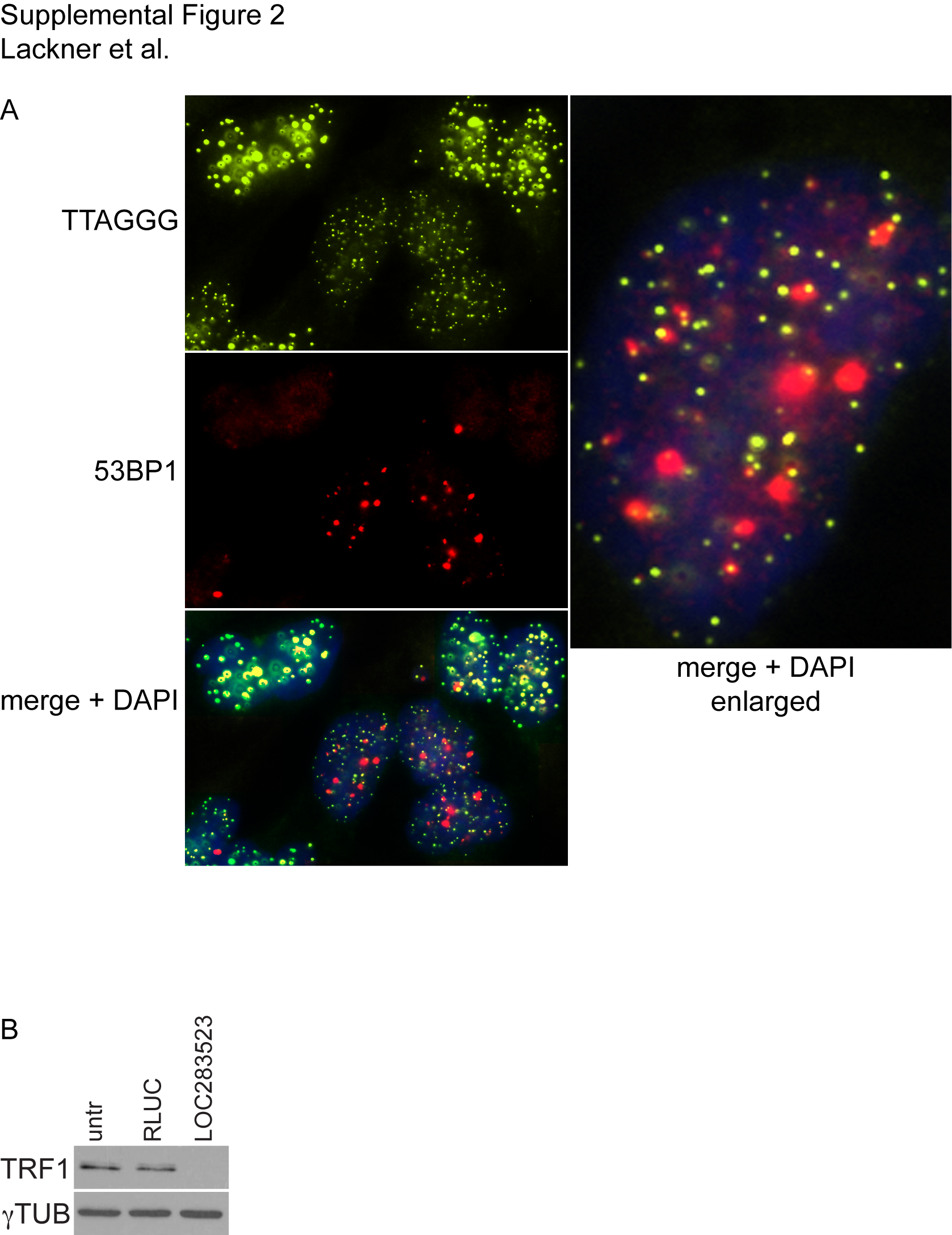

Supplement: Figure S2 — (A) Spontaneous TIF formation in control cells. IF-FISH analysis of cells in RLUC-transfected control cells. Telomeres were detected by hybridization to a FITC coupled TTAGGG probe and damage foci were visualized by 53BP1 staining. (B) Targeting LOC283523 reduces TRF1 expression. Western analysis of TRF1 expression after transfection with siGENOME pool against LOC283523 72 hrs post transfection.gamma-Tubulin serves as loading control. (TIF) [file pone.0021407.s002.tif]
