# Supplementary material for: A siRNA-Based Screen for Genes Involved in Chromosome End Protection
Source: PLoS One. 2011 Jun 23;6(6):e21407. doi: 10.1371/journal.pone.0021407 (PMC3121770; doi:10.1371/journal.pone.0021407)
Supplement: Table S1 — List of all candidate genes tested in the screen. No data were obtained for the genes indicated in red due to technical problems. (DOCX) [file pone.0021407.s003.docx]

| **Table S1. List of all candidate genes tested in the screen.** | |
| --- | --- |
| **Screen ID** | **Gene Symbol** |
| K1 | AAK1 |
| K2 | CHEK1 |
| K3 | CHKA |
| K4 | AKAP13 |
| K5 | AKT3 |
| K6 | CDC7 |
| K7 | CDK5R2 |
| K8 | COPB2 |
| K9 | CSNK1D |
| K10 | DLG4 |
| K11 | EKI1 |
| K12 | FASTK |
| K13 | FGFR4 |
| K14 | MASTL |
| K15 | FUK |
| K16 | GALK2 |
| K17 | NTRK3 |
| K18 | PAK6 |
| K19 | PLK1 |
| K20 | PPP4C |
| K21 | PRKX |
| K22 | PXK |
| K23 | TEX14 |
| K24 | TRIB2 |
| K25 | TTK |
| R1 | CALCRL |
| R2 | EMR3 |
| R3 | GNAZ |
| R4 | GPR77 |
| R5 | LTB4R2 |
| R6 | NPY1R |
| R7 | OR7A17 |
| D1 | CHAF1A |
| D2 | CLSPN |
| D3 | ORC6L |
| D4 | PRPF8 |
| D5 | RAD23A |
| D6 | SF3A1 |
| D7 | SFPQ |
| D8 | XPA |
| D9 | BRF1 |
| D10 | FOXO1A |
| D11 | MITF |
| D12 | MYBL2 |
| D13 | MYC |
| D14 | SKIIP |
| D15 | SSX1 |
| D16 | ZHX2 |
| D17 | ALDOA |
| D18 | P4HB |
| D19 | PA2G4 |
| D20 | PDE3B |
| D21 | PDHB |
| D22 | PHPT1 |
| D23 | POLR2D |
| D24 | PRODH2 |
| D25 | PSMB4 |
| D26 | PSMB6 |
| D27 | PSMB9 |
| D28 | RDH5 |
| D29 | RRM1 |
| D30 | RRM2 |
| D31 | UBE2E3 |
| D32 | TOP2A |
| D33 | SERTAD3 |
| D34 | DCLRE1A |
| D35 | ANP32B |
| D36 | MYBBP1A |
| D37 | PCNA |
| D38 | POLA |
| D39 | POLE |
| D40 | PSMA1 |
| D41 | REV3L |
| D42 | TOPBP1 |
| D43 | U5-200KD |
| D44 | SUPT6H |
| D45 | C13ORF22 |
| D46 | TNFAIP3 |
| D47 | USP1 |
| D48 | USP10 |
| D49 | USP19 |
| D50 | BARD1 |
| D51 | FBXL20 |
| D52 | UBE3A |
| D53 | RBBP6 |
| D54 | WHSC1L1 |
| D55 | MLL2 |
| D56 | BRCA1 |
| D57 | FBXO5 |
| D58 | RAD51 |
| D59 | WHSC1 |
| D60 | NFX1 |
| D61 | CENPE |
| D62 | MGC26484 |
| D63 | PPM1B |
| D64 | PPP2R1B |
| D65 | PPP2R5D |
| D66 | PTPDC1 |
| D67 | PTPN23 |
| D68 | PPM1A |
| D69 | LAMR1 |
| D70 | IK |
| D71 | LDLR |
| D72 | ARNT2 |
| D73 | CRSP6 |
| D74 | CRSP7 |
| D75 | PLRG1 |
| D76 | PPARBP |
| D77 | PPARD |
| D78 | RARA |
| D79 | THRAP3 |
| D80 | DAP3 |
| D81 | UBA52 |
| D82 | NEB |
| D83 | NHP2L1 |
| D84 | RPS5 |
| D85 | TAF1C |
| D86 | MOBK1B |
| D87 | ATRX |
| D88 | YAF2 |
| D89 | DDB1 |
| D90 | DKFZP451J0118 |
| D91 | CASP8AP2 |
| D92 | CCNA2 |
| D93 | CCNK |
| D94 | E2F3 |
| D95 | EIF3S10 |
| D96 | ERH |
| D97 | FLJ10665 |
| D98 | GLTSCR2 |
| D99 | HIP1 |
| D100 | HIRIP3 |
| D101 | FBXO31 |
| D102 | HMGN1 |
| D103 | HNRPK |
| D104 | FAM35A |
| D105 | NFKBIL1 |
| D106 | NOL1 |
| D107 | NUSAP1 |
| D108 | OBRGRP |
| D109 | PELP1 |
| D110 | PLA2R1 |
| D111 | PSMD7 |
| D112 | PSMD8 |
| D113 | MPST |
| D114 | NDE1 |
| D115 | NDEL1 |
| D116 | RPL10 |
| D117 | RPL11 |
| D118 | RPL32 |
| D119 | RPL5 |
| D120 | RPS16 |
| D121 | SART1 |
| D122 | SNIP1 |
| D123 | SNN |
| D124 | SSSCA1 |
| D125 | SUPT5H |
| D126 | TAF4 |
| D127 | RAMP |
| D128 | RBBP5 |
| D129 | RBBP8 |
| D130 | XPO1 |
| D131 | XRCC4 |
| G1 | GCN5L2 |
| G2 | HNRPF |
| G3 | HNRPU |
| G4 | NAP1L2 |
| G5 | NPAT |
| G6 | NUP88 |
| G7 | PMS2L1 |
| G8 | PRH1 |
| G9 | AF15Q14 |
| G10 | AD023 |
| G11 | PSMA7 |
| G12 | RFC3 |
| G13 | RPA2 |
| G14 | TDG |
| G15 | FAM16AX |
| G16 | RBM10 |
| G17 | AQR |
| G18 | VPRBP |
| G19 | SAE1 |
| G20 | DSCR4 |
| G21 | ZNF272 |
| G22 | MYST2 |
| G23 | WDHD1 |
| G24 | NRM |
| G25 | KIAA1002 |
| G26 | KIAA1018 |
| G27 | KIAA1117 |
| G28 | KIAA0261 |
| G29 | KIAA0179 |
| G30 | KIAA0553 |
| G31 | FCHO1 |
| G32 | C19ORF7 |
| G33 | KIAA0841 |
| G34 | SIRT5 |
| G35 | ORC3L |
| G36 | DKFZP564J0123 |
| G37 | KIAA0794 |
| G38 | SENP6 |
| G39 | DKFZP434L187 |
| G40 | DKFZP434J154 |
| G41 | SND1 |
| G42 | EPPB9 |
| G43 | ZNF364 |
| G44 | NOSIP |
| G45 | HSU79274 |
| G46 | STRN3 |
| G47 | ZNF295 |
| G48 | RNF141 |
| G49 | MED31 |
| G50 | PNAS-4 |
| G51 | CGI-62 |
| G52 | NAT5 |
| G53 | NY-REN-58 |
| G54 | COPS4 |
| G55 | C15ORF15 |
| G56 | ZNF639 |
| G57 | C14ORF87 |
| G58 | LOC51234 |
| G59 | MGC12197 |
| G60 | C13ORF12 |
| G61 | POLK |
| G62 | C9ORF114 |
| G63 | C14ORF166 |
| G64 | NYREN18 |
| G65 | WBP11 |
| G66 | HBXAP |
| G67 | PCBP3 |
| G68 | FBXW5 |
| G69 | FLJ20010 |
| G70 | LOC54499 |
| G71 | KIAA1193 |
| G72 | FLJ20729 |
| G73 | KIAA1221 |
| G74 | DKFZP434I1117 |
| G75 | ZRANB1 |
| G76 | FLJ20014 |
| G77 | FLJ20582 |
| G78 | FBXO34 |
| G79 | KIF26B |
| G80 | C10ORF118 |
| G81 | FLJ10204 |
| G82 | FLJ20850 |
| G83 | FLJ10260 |
| G84 | FLJ10330 |
| G85 | TRIM68 |
| G86 | FLJ10379 |
| G87 | FLJ10385 |
| G88 | CDCA8 |
| G89 | HRMT1L6 |
| G90 | C14ORF104 |
| G91 | RIF1 |
| G92 | FLJ10652 |
| G93 | RCBTB1 |
| G94 | FLJ10748 |
| G95 | SMU1 |
| G96 | FLJ10826 |
| G97 | PHF10 |
| G98 | FLJ11151 |
| G99 | FLJ11196 |
| G100 | MCM10 |
| G101 | OTUD5 |
| G102 | SUHW3 |
| G103 | C9ORF86 |
| G104 | RNF20 |
| G105 | DJ971N18.2 |
| G106 | FLJ20257 |
| G107 | BEXL1 |
| G108 | DHX33 |
| G109 | C5ORF15 |
| G110 | DDX24 |
| G111 | KIAA0493 |
| G112 | KIAA1160 |
| G113 | KIAA1210 |
| G114 | KIAA1573 |
| G115 | ZSWIM6 |
| G116 | C6ORF47 |
| G117 | ZNF77 |
| G118 | C12ORF14 |
| G119 | ZNF335 |
| G120 | INF2 |
| G121 | ZNF574 |
| G122 | MGC2494 |
| G123 | MGC2654 |
| G124 | MGC2655 |
| G125 | FLJ13119 |
| G126 | ROGDI |
| G127 | DHX40 |
| G128 | FLJ21816 |
| G129 | C10ORF68 |
| G130 | FLJ14007 |
| G131 | C5ORF14 |
| G132 | FLJ13984 |
| G133 | JMJD5 |
| G134 | FLJ13150 |
| G135 | C9ORF82 |
| G136 | FLJ13941 |
| G137 | PHF17 |
| G138 | ZNF442 |
| G139 | KIAA1718 |
| G140 | C12ORF22 |
| G141 | MGC2963 |
| G142 | KIAA1786 |
| G143 | MCM8 |
| G144 | MGC13125 |
| G145 | ZNF496 |
| G146 | FLJ14800 |
| G147 | MUM1 |
| G148 | BJ-TSA-9 |
| G149 | MGC15737 |
| G150 | C20ORF99 |
| G151 | TNKS1BP1 |
| G152 | C14ORF152 |
| G153 | MGC15407 |
| G154 | C7ORF30 |
| G155 | MGC15763 |
| G156 | LOC92129 |
| G157 | LOC92312 |
| G158 | LOC92558 |
| G159 | ASB16 |
| G160 | PP3856 |
| G161 | C21ORF66 |
| G162 | C14ORF28 |
| G163 | C14ORF8 |
| G164 | MGC35048 |
| G165 | LOC124751 |
| G166 | C19ORF21 |
| G167 | FLJ32112 |
| G168 | C20ORF152 |
| G169 | LOC145741 |
| G170 | LOC147991 |
| G171 | FLJ32421 |
| G172 | FLJ31821 |
| G173 | KIAA1754L |
| G174 | MGC22679 |
| G175 | ZNF567 |
| G176 | LOC200008 |
| G177 | ZNRF2 |
| G178 | C6ORF167 |
| G179 | GOR |
| G180 | CASC2 |
| G181 | MGC39696 |
| G182 | ZNF619 |
| G183 | DKFZP313N0621 |
| G184 | FLJ37673 |
| G185 | LOC285733 |
| G186 | LOC286235 |
| G187 | FLJ37659 |
| G188 | KIAA1875 |
| G189 | DKFZP686O1689 |
| G190 | ZNF81 |
| G191 | FLJ39963 |
| G192 | DKFZP434K1323 |
| G193 | FLJ26056 |
| G194 | C6ORF204 |
| G195 | FLJ40008 |
| G196 | FLJ41841 |
| G197 | LOC401610 |
| G198 | LOC283523 |
| G199 | LOC122706 |
| G200 | LOC220717 |
| G201 | LOC285047 |
| G202 | LOC151443 |
| G203 | GABRR3 |
| G204 | LOC347544 |
| G205 | LOC158345 |
| G206 | LOC283247 |
| G207 | LOC91561 |
| G208 | LOC90193 |
| G209 | LOC400743 |
| G210 | LOC400793 |
| G211 | LOC389452 |
| G212 | LOC389506 |
| G213 | LOC401400 |
| G214 | LOC389727 |
| G215 | LOC399937 |
| G216 | LOC399940 |
| G217 | LOC390299 |
| G218 | LOC387991 |
| G219 | LOC388339 |
| G220 | LOC390980 |
| G221 | ZNF429 |
| G222 | FLJ16139 |
| G223 | MGC17624 |
