# Supplementary material for: A siRNA-Based Screen for Genes Involved in Chromosome End Protection
Source: PLoS One. 2011 Jun 23;6(6):e21407. doi: 10.1371/journal.pone.0021407 (PMC3121770; doi:10.1371/journal.pone.0021407)
Supplement: Table S2 — Repetitions of suppression of 11 candidate factors. (DOCX) [file pone.0021407.s004.docx]

| **Table S2. Repetitions of suppression of 11 candidate factors.** | | | | |
| --- | --- | --- | --- | --- |
| **Repeat 1** |  |  |  |  |
| **Screen ID** | **Gene symbol** | **# TIF** | **Stdev** | **Remarks** |
| Control | Untransfected | 0.2 | 0.4 |  |
| Control | RLUC siRNA | 0.3 | 0.4 |  |
| Control | TRF2 OTP siRNA pool | 8.4 | 6.5 |  |
| G198 | LOC283523 | 15.3 | 8.7 | siRNA pool also targets TRF1 |
| D83 | NHP2L1 | 1.1 | 1.1 | many 53BP1 foci, few cells |
| D29 | RRM1 | 1.2 | 1.2 | many 53BP1 foci, few cells |
| G122 | MGC2494 | 1.8 | 2.3 | many 53BP1 foci |
| G144 | MGC13125 | ND | ND | broken coverslip |
| D37 | PCNA | 1.0 | 1.1 | many 53BP1 foci |
| D88 | YAF2 | 0.2 | 0.4 | no phenotype |
| D38 | POLA | 2.5 | 3.4 | many 53BP1 foci |
| D14 | SKIIP | 1.7 | 1.6 | many 53BP1 foci, very few cells |
| D6 | SF3A1 | 2.3 | 2.4 | many 53BP1 foci |
| D89 | DDB1 | 1.9 | 1.9 | many 53BP1 foci |
|  |  |  |  |  |
| **Repeat 2** |  |  |  |  |
| **Screen ID** | **Gene symbol** | **# TIF** | **Stdev** | **Remarks** |
| Control | Untransfected | 0.1 | 0.4 |  |
| Control | RLUC siRNA | 0.3 | 0.5 |  |
| Control | TRF2 OTP siRNA pool | 5.2 | 5.1 |  |
| G198 | LOC283523 | 9.6 | 10.5 | siRNA pool also targets TRF1 |
| D83 | NHP2L1 | 4.3 | 2.0 | many 53BP1 foci, few cells |
| D29 | RRM1 | 3.2 | 2.3 | many 53BP1 foci, few cells |
| G122 | MGC2494 | 1.7 | 2.3 | many 53BP1 foci |
| G144 | MGC13125 | 3.6 | 2.7 | many 53BP1 foci |
| D37 | PCNA | 2.7 | 2.1 | many 53BP1 foci |
| D88 | YAF2 | 0.5 | 0.7 | no phenotype |
| D38 | POLA | 3.2 | 2.1 | many 53BP1 foci |
| D14 | SKIIP | 2.5 | 2.5 | many 53BP1 foci, few cells |
| D6 | SF3A1 | 3.8 | 4.3 | many 53BP1 foci |
| D89 | DDB1 | 3.4 | 2.8 | many 53BP1 foci |
